# Elastin-derived peptides enhance melanoma growth *in vivo* by upregulating the activation of Mcol-A (MMP-1) collagenase

**DOI:** 10.1038/sj.bjc.6605926

**Published:** 2010-10-19

**Authors:** J Devy, L Duca, B Cantarelli, D Joseph-Pietras, A Scandolera, A Rusciani, L Parent, J Thevenard, S Brassart Pasco, M Tarpin, L Martiny, L Debelle

**Affiliations:** 1 Université de Reims Champagne Ardenne (URCA), Laboratoire SiRMa, UMR CNRS 6237, IFR 53 Interactions cellules microenvironment, UFR Sciences Exactes et Naturelles, Moulin de la Housse, BP 1039, 51687 Reims Cedex 2, France; 2 Genetic Vaccine Group, Tenovus Laboratory, Cancer Sciences Division, School of Medicine, Southampton University Hospitals, Southampton, UK; 3 Laboratoire de Biochimie Médicale et Biologie Moléculaire, UMR CNRS 6237, UFR Médecine, 51 rue Cognacq Jay, 51095 Reims Cedex, France

**Keywords:** elastin-derived peptides, melanoma, *in vivo*, Mcol-A, predictive factor

## Abstract

**Background:**

Elastin peptides possess several biological activities and *in vitro* data suggest they could be involved in the early phase of melanoma growth.

**Methods:**

Using diverse *in vitro* and *in vivo* techniques (cell proliferation, invasion and migration assays, zymography, western blots, collagen degradation assay, reverse transcription PCR, melanoma allographs and immunohistochemistry), we analysed the effect of elastin-derived peptides (EDPs) on B16F1 melanoma growth and invasion, as well as on the proteolytic systems involved.

**Results:**

We found that EDPs dramatically promote *in vivo* tumour development of B16F1 melanoma, as well as their *in vitro* migration and invasion. The inhibition of serine proteases and matrix metalloproteinases (MMPs) activities, by aprotinin and galardin, respectively, demonstrated that these enzymes were involved in these processes. However, we found that EDPs did not increase urokinase-type plasminogen activator, tissue-type plasminogen activator or MMP-2 expression and/or activation, neither *in vitro* nor *in vivo*. Nevertheless, we observed a strong increase of pro-MMP-9 secretion in EDPs-treated tumours and, more importantly, an increase in the expression and activation of the murine counterpart of MMP-1, named murine collagenase-A (Mcol-A). Moreover, we show that plasminogen system inhibition decreases collagen degradation by this enzyme. Finally, the use of a specific blocking antibody against Mcol-A abolished EDP-induced B16F1 invasion *in vitro*, showing that this MMP was directly involved in this process.

**Conclusion:**

Our data show that *in vivo*, EDPs are involved in melanoma growth and invasion and reinforced the concept of elastin fragmentation as a predictive factor.

Extracellular matrix (ECM) degradation is a prerequisite for cancer progression. Beside the fact that its degradation is essential to allow cell migration through its three-dimensional architecture, the generated ECM fragments are not inactive. Indeed, ECM proteolysis leads to the release of matrikines, matrix fragments that display proper biological activities from their original matrix counterparts (Hornebeck *et al*, 2002).

Elastin constitutes one of the ECM proteins responsible for the elasticity of tissues (Kielty *et al*, 2002). Protease-driven elastin degradation can occur during physiopathological processes such as cancer progression (Hornebeck *et al*, 2005). The generated bioactive elastin-derived peptides (EDPs) are thought to contribute to tumour progression (Duca *et al*, 2004).

Several *in vitro* studies (Yusa *et al*, 1989; Timar *et al*, 1991; Svitkina and Parsons, 1993; Ntayi *et al*, 2004) suggest that EDPs could promote cutaneous melanoma growth and invasion. For instance, it has been shown that the incidence of metastases increased as melanoma reached the subepidermal and dermal layers, suggesting that elastin degradation could facilitate melanoma progression (Breslow, 1970). In addition, intense elastin fragmentation was detected at the *in vivo* invasive front of melanoma (Ntayi *et al*, 2004). It has moreover been shown that EDPs amplify melanoma invasion through their binding to the elastin-binding protein, *α*_v_*β*_3_ and galectin-3 receptors (Pocza *et al*, 2008). However, no *in vivo* studies were carried out to determine the influence of EDPs on melanoma growth and invasion.

Cutaneous melanoma is characterised by its strong ability to form metastases (Hofmann *et al*, 2005). If melanoma is diagnosed early, it can be cured by surgical resection. On the other hand, metastatic malignant melanoma is refractory to existing therapies and has a very poor prognosis. New treatment strategies are consequently urgently needed and the identification of new early predictive marker of melanoma metastatic potential is of crucial interest to handle the pathology as fast as possible.

Two classes of proteinases have been shown to be involved in melanoma invasion: the matrix metalloproteinases (MMPs) and the plasminogen activation system (Pasco *et al*, 2004a).

The MMPs are involved in the degradation of the ECM both in physiological and physiopathological conditions. Human melanoma invasion involved MMP-1, MMP-2, MMP-9 and MT1-MMP (Walker and Woolley, 1999; Hofmann *et al*, 2005). Nevertheless, and on the contrary of the others precited MMPs, MMP-1 was initially thought to be absent from murine tissues. However, murine collagenase-A (Mcol-A) was recently discovered. It has a role similar to human MMP-1, and is able to degrade fibrillar collagens (Balbin *et al*, 2001). The MMPs are synthesised as inactive zymogens and their proteolytic activation occurs in the extracellular space by others MMPs or by plasmin.

The plasminogen/plasmin system has largely been documented and is involved in melanoma invasion (de Vries *et al*, 1996; Andreasen *et al*, 1997, 2000). The cleavage of plasminogen involves two activators named urokinase-type plasminogen activator (uPA) and tissue-type plasminogen activator (tPA).

We show, for the first time, that EDPs strongly enhance murine melanoma development *in vivo*. This important finding point out that elastin degradation, which occurs during melanoma development, is one of the events that could drive tumour growth. Moreover, using several biochemical methods, we demonstrate that EDPs specifically enhance *in vitro* and *in vivo* Mcol-A expression and that this enzyme could be responsible for EDP-stimulated melanoma invasion.

In conclusion, our results show that elastin degradation products are important modulators of *in vivo* melanoma growth and invasion through Mcol-A (MMP-1) expression. Moreover, our work demonstrates that elastin degradation could be an important predictive factor of evolution in melanoma.

## Materials and methods

### Reagents and antibodies

Elastin-derived peptides (*κ*-elastin, also noted as EDPs) were prepared as previously described (Debelle *et al*, 1995; Brassart *et al*, 2001). Briefly, insoluble elastin was prepared from bovine ligamentum nuchae by hot alkali treatment. Its purity was assessed by comparing its amino acid composition to that predicted from the elastin gene product. Soluble elastin peptides were obtained from insoluble elastin by organo-alkaline hydrolysis. This was achieved using 1 M KOH in 80% aqueous ethanol. The obtained mixture of elastin peptides is termed *κ*-elastin and exhibits the same biological and physical properties than elastin hydrolysates (Brassart *et al*, 2001). Bovine serum albumin (BSA), gelatin, plasminogen and aprotinin, a commonly used inhibitor of the plasminogen system (Ramont *et al*, 2003), were purchased from Sigma (Saint-Quentin Fallavier, France). All reagents for cell culture were from Gibco BRL (Invitrogen, Cergy Pontoise, France). Galardin, a synthetic broad-spectrum MMP inhibitor (Agren *et al*, 2001), the anti-human-MMP1 (Ab-3) used for western blot analyses and the anti-human-MMP-1 (Ab-6) used in blocking experiments (Brennan *et al*, 2003) were from Calbiochem (distributed by VWR Int., Strasbourg, France). All these antibodies were checked for their mouse cross-reactivity. The anti-monocyte/macrophage (MoMa-2) was from BMA Biomedicals (Rheinstrasse, Switzerland). All other reagents were from Sigma.

### Cell culture and treatments

B16F1 cells, a lung metastatic subline of murine B16 melanoma, were cultured in RPMI 1640 medium supplemented with 10% heat-inactivated fetal bovine serum (FBS) in 25 cm^2^ flasks at 37°C in a humidified atmosphere of 5% CO_2_/95% air. For the evaluation of EDPs effects on proteases expression and activation, cells were seeded onto 24-well plates and subconfluent cultures were washed twice in phosphate-buffered saline to eliminate residual FBS and then incubated or not with 50 *μ*g ml^–1^ EDPs for 48 h. After the incubation period, the culture media were harvested, centrifuged for 10 min at 500 **g** to eliminate cellular debris and then submitted to western blot or zymography analyses. In all experiments, cell viability was >95% when cell culture supernatants were collected as evaluated by MTT assay. For cell proliferation assay, B16F1 were seeded in 96-well plates in complete medium with or without EDPs for 72 h. Cell proliferation was determined by counting cells with a Malassez cell.

### Migration assay

B16F1 cells (75.10^5^) have been seeded in 12-well plates in complete medium. After 48 h, cell media were eliminated, and a homogeneous wound was created in each well by scraping cells with a tip followed by two washings with RPMI 1640 in order to eliminate dead cells and cellular debris. Cells were stimulated or not for 48 h with 50 *μ*g ml^–1^ EDPs and cell migration was evaluated by videomicroscopy (Axiovert 200M, Zeiss, Heidelberg, Germany).

### *In vitro* invasion assays

The invasive potential of tumour cells was examined using modified Boyden chambers, 6.5 mm diameter and 8 *μ*m pore (Transwell, Costar, Brumath, France) according to the manufacturer's instructions. Briefly, cells were suspended in serum-free RPMI containing 0.2% BSA, and 100 *μ*l of the cell suspension (50 × 10^3^ cells) was seeded onto the upper compartment of the Transwell coated with Matrigel (40 *μ*g per well). In the lower compartment, 800 *μ*l RPMI containing 10% FBS and 2% of BSA was used as chemoattractant. The cells were incubated with or without aprotinin (100 *μ*g ml^–1^), galardin (10^−9^ M), both or with a monoclonal anti-MMP-1-blocking antibody (10 *μ*g ml^–1^) and stimulated or not with EDPs (50 *μ*g ml^–1^). Transwells were incubated at 37°C under 5% CO_2_/95% air atmosphere for 40 h and cells were then fixed with methanol. Non-invading cells remaining on the upper side of the filter were scrapped off. Invading cells on the lower side of the filter were then stained with crystal violet for 15 min, microscopically observed and counted in five fields at × 200 magnification. The invasive activity of cancer cells was expressed as the mean number of cells that have crossed Matrigel.

### Mice and tumours

Female C57Bl6 mice (average body weight: 18–20 g) were purchased from Harlan France (Gannat, France). Animals were individually caged and given food and water *ad libitum*. They were kept in a room with constant temperature and humidity. All mice were acclimatised to laboratory conditions for 1 week before starting the experiments. The experiments were conducted according to the recommendations of the Centre National de la Recherche Scientifique. Mice (five mice per group) were injected subcutaneously with 2.5 × 10^5^ B16F1 cells in 100 *μ*l of RPMI containing or not EDPs (100 *μ*g). The mice were reinjected at tumour site with EDPs at day 7. Each tumour was measured according to *v*=1/2 × A^2^ × B (A, smallest superficial diameter; B, largest superficial diameter) (Pasco *et al*, 2004b).

### Tumours and cell protein extraction

Tumours were surgically extracted at day 10 and homogenised in Net Buffer (100 mM Tris, pH 7.4, 300 mM NaCl, 10 mM EDTA, 2% NP40). For cell extracts, cells were homogenised in lysis buffer (Tris 10 mM, pH 7.4, NaCl 150 mM, 1% Triton X-100, EDTA 5 mM) containing protease inhibitors. Proteins were quantified using the Bradford method, with BSA as a standard.

### Immunohistochemical studies

Sections of 6 *μ*m from formalin-fixed, paraffin-embedded tumours were used for immunohistochemical detection of monocyte/macrophage and Mcol-A. The sections were deparaffinised, rehydrated and treated with citrate buffer for antigen retrieval. Endogenous peroxidase activity was blocked with incubation of the slides in 3% H_2_O_2_ in phosphate-buffered saline for 10 min. Sections were preincubated for 30 min with 1% BSA and incubated overnight at 4°C with a sheep anti-MMP-1 antibody (1/100) or rat anti-monocyte/macrophage antibody (1/50) followed by an incubation with the corresponding secondary antibody linked to peroxidase treatment. Antibody binding was visualised with diaminobenzidin, and then the slides were counterstained with Methylene Green zinc chloride double salt (Sigma).

### Western blot analysis

Protein of 20 *μ*g was subjected to SDS–polyacrylamide gel electrophoresis and transferred onto nitrocellulose membranes. Membranes were incubated in blocking buffer (5% non-fat dry milk, 0.1% Tween 20 in 50 mM Tris–HCl buffer, 150 mM NaCl, pH 7.5) for 1 h at room temperature. They were thereafter incubated overnight at 4°C with the rabbit anti-MMP-1 (1/500) primary antibody diluted in blocking buffer and then with the corresponding peroxidase-conjugated secondary antibody for 1 h at room temperature. Immune complexes were visualised using enhanced chemiluminescence.

### Gelatin and gelatin plasminogen zymography

Gelatinases and plasminogen activators (PAs) expression from conditioned media of B16F1 and tumour extracts (10 *μ*g proteins per sample) were analysed using SDS–polyacrylamide gel electrophoresis containing 0.1% gelatin for gelatin zymography or 0.1% of gelatin and 0.25 U ml^–1^ plasminogen for gelatin–plasminogen zymography. After electrophoresis, the gels were soaked in 2.5% (v/v) Triton X-100 solution for 1 h to remove SDS. For gelatin zymography, gels were then incubated in 50 mM Tris–HCl (pH 7.6) containing 5 mM CaCl_2_ and 200 mM NaCl overnight at 37°C. For gelatin–plasminogen zymography, gels were then incubated overnight at 37°C in 100 mM glycine buffer, 5 mM EDTA, pH 8.0. The gels were then stained with 0.1% (w/v) G250 Coomassie brilliant blue in 45% (v/v) methanol/10% (v/v) acetic acid. After 10 min, the gels were destained with 25% (v/v) methanol/10% acetic acid for 1 h. The proteolytic activity was detected as clear bands on a blue background of the Coomassie brilliant blue-stained gel.

### Reverse transcription PCR

Total RNA was isolated after lysis of B16F1 cells. Following OD assessment and gel analysis, 250 ng total RNA was reverse transcribed. The following cycling profile was used: 30 s at 94°C, 30 s at 57°C, 30 s at 72°C for 30 cycles. The PCR products were analysed in 2% ethidium bromide-stained agarose gels. The forward (F) and reverse (R) primer sequences for the different PCR products and their size in base pairs (bp) were as follows:

Mcol-A F: 5′-TTCATGCCAGAACCTGAGCTC-3′,

Mcol-A R: 5′-GGGAAGCCAAAGAAACTGTGG (110 bp)-3′

*β*- Actin F: 5′-GTGTGACGTGGACATCCGC-3′,

*β*-Actin R: 5′-CTGCATCCTGTCGGCAATG-3′ (91 bp).

### Collagen degradation assay

The influence of Mcol-A expression following EDPs treatment on collagen degradation was evaluated using DQ-collagen (Invitrogen) following the manufacturer's instructions. Briefly, 100 *μ*l from B16F1 conditioned media (48 h) incubated or not with EDPs (50 *μ*g ml^–1^) and/or aprotinin (100 *μ*g ml^–1^) were incubated with reaction buffer (80 *μ*l) and 20 *μ*l of DQ-collagen (100 *μ*g ml^–1^) for 4 h. The degradation of DQ-collagen was evaluated by fluorescence emission (*λ*_ex_=495 nm; *λ*_em_=515 nm).

### Densitometric analysis and statistical evaluation

Each band from polyacrylamide gels was quantified using Quantity One software (Bio-Rad Laboratories, Marne-la-Vallée, France). Each experiment was performed at least three times, from separate sets of culture. Data were expressed as mean±s.e.m. Comparisons were performed using Student's *t*-test.

## Results

### EDPs enhance melanoma growth *in vivo* and *in vitro* cell proliferation

**Figure 1:**
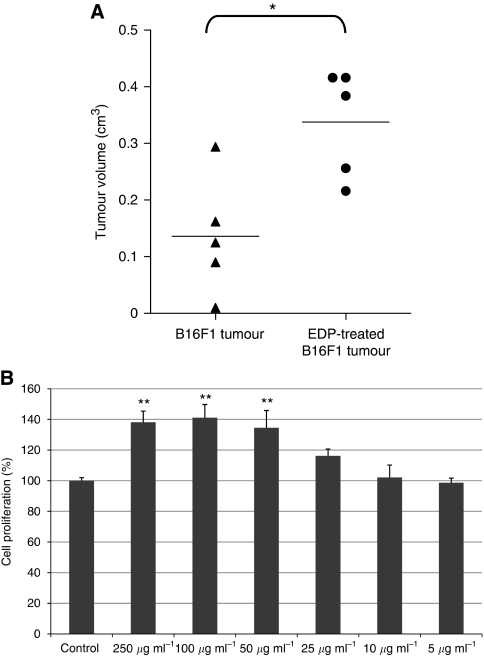
Elastin-derived peptides increase melanoma tumour growth *in vivo* (**A**) and *in vitro* cell proliferation (**B**). (**A**) B16F1 or EDP-pretreated B16F1 were subcutaneously injected to female syngenic C57Bl6 mice (2.5 × 10^5^ cells per mouse) as described in the Materials and Methods section. Mice were killed at day 10. Tumour sizes were measured and statistical significance was calculated according to the Student's *t*-test. Data represent the average of five mice. A second experiment conducted under the same conditions gave identical results. ^*^Significantly different at *P*<0.05. (**B**) B16F1 were seeded in 96-well plates in complete medium with or without EDPs for 72 h. Cell proliferation was determined by counting cells with a Malassez cell. Data are expressed as mean±s.e.m. values from three independent experiments, each performed in triplicate. ^**^Significantly different at *P*<0.01.

To determine the effect of EDPs on melanoma development, control B16F1 cells or B16F1 preincubated for 30 min in the presence of EDPs (100 *μ*g) were subcutaneously injected into the right side of syngenic C57Bl6 mice. Control and EDP-treated mice were killed on day 10. Tumours were excised and measured. Tumour mean volume was increased by at least three-fold (*P*<0.05) in EDP-treated mice *vs* control (Figure 1A), showing that EDPs strongly enhanced melanoma growth *in vivo*. To confirm that such an effect is directly mediated by the elastin peptides, that is, that EDPs do not signal via other ECM components, we evaluate their pro-proliferative abilities towards B16F1 cells. The stimulation of melanoma cells with EDPs in the presence of 10% FBS triggered cell proliferation at 72 h in a dose-dependent manner (Figure 1B). The optimal concentration is reached since 50 *μ*g ml^–1^. It has to be emphasised that EDPs present no effect on cell proliferation for FBS concentrations inferior to 10%. These results consequently demonstrate that EDPs directly enhanced melanoma growth.

### EDPs increase B16F1 *in vitro* migration and invasion

**Figure 2:**
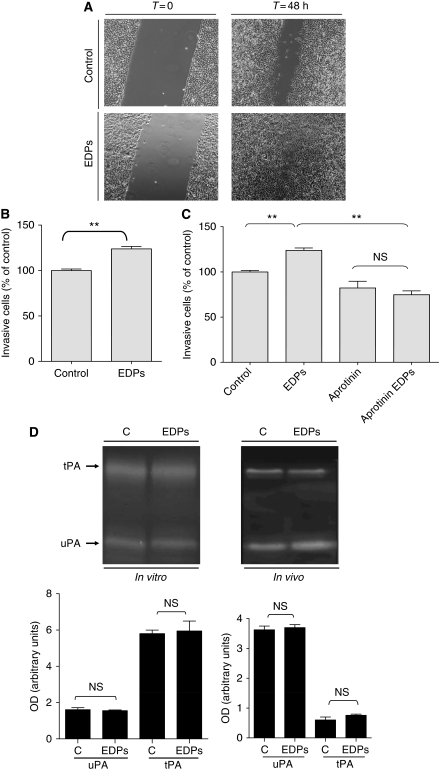
Elastin-derived peptides stimulate B16F1 migration (**A**) and invasion (**B**). Invasion involves the plasminogen system (**C**) without inducing tPA or uPA (**D**). (**A**) B16F1 cells have been seeded in 12-well plates and a homogeneous wound was created in each well by scraping cells with a tip. Cells were stimulated or not for 48 h with 50 *μ*g ml^–1^ EDPs and cell migration was evaluated by videomicroscopy. (**B**) Cellular invasive potential was assayed using Transwell coated with Matrigel (40 *μ*g per well). In total, 50 × 10^3^ cells in 100 *μ*l of RPMI 1640 with or without EDPs (50 *μ*g ml^–1^) were deposited into the upper chamber. The lower chamber contained 10% FBS and 2% of BSA. Incubation was for 40 h. Data are expressed as mean±s.e.m. values from three independent experiments, each performed in triplicate. ^**^Significantly different at *P*<0.01. (**C**) is same as (**B**) except for the presence or not of aprotinin (100 *μ*g ml^–1^). ^**^Significantly different at *P*<0.01. NS=nonsignificantly different. (**D**) Upper panel: Gelatin plasminogen zymography was performed as described in the Materials and Methods section. The gels presented are representative of several *in vitro* (*n*=3) and *in vivo* (*n*=5) experiments. Lower panel: Quantification of *in vitro* and *in vivo* expressions of tPA and uPA was carried out using densitometry and calculating using Quantity One Software. C=untreated control; EDPs=EDP-treated cells or tumours; NS=nonsignificantly different.

B16F1 cells were tested for their ability to migrate following EDPs stimulation in an *in vitro* system. Briefly, B16F1 were seeded on plastic dishes then scrapped at the centre of the dish. The migration of the cells was evaluated by videomicroscopy using their ability to fill the gap. These experiments were performed using 2.5% FBS, a concentration for which EDPs has no effect on cell proliferation. We found that EDPs enhanced B16F1 migration as the artificially created wound was more filled compared with control (Figure 2A). B16F1 cells were then tested for their ability to migrate through Matrigel-coated (40 *μ*g per well) membranes for 40 h in the presence or absence of EDPs. The incubation of melanoma cells with EDPs led to a significant increase in their invasive properties (+36%) comparatively to control cells (Figure 2B). These results demonstrated that EDPs increased melanoma cell invasion and showed that, in addition to trigger *in vivo* growth, they enhance cell invasion. These results support the detrimental role of EDPs in *in vivo* melanoma development.

### Aprotinin decreases invasion of EDP-induced B16F1 cells

One of the proteolytic systems involved in melanoma invasion is the PA system (de Vries *et al*, 1996; Andreasen *et al*, 1997, 2000). In order to determine the contribution of PA in EDP-induced B16F1 melanoma invasion, the invasiveness of B16F1 or EDP-treated B16F1 cells was assayed *in vitro* in the presence of aprotinin (Ramont *et al*, 2003). As shown in Figure 2C, aprotinin inhibited invasion of unstimulated or EDP-stimulated B16F1 cells. It is however important to note that the increase in invasion observed when cells are treated with EDPs is no longer observed, demonstrating that the increase involved the plasminogen system.

To analyse the effects of EDPs on PAs uPA and tPA, we performed gelatin plasminogen zymography from tumour lysates and conditioned culture media. No increase in uPA nor tPA expression was observed neither *in vivo* nor *in vitro* (Figure 2D). Thus, we hypothesised that EDPs could enhance melanoma invasion by inducing pro-MMPs expression, whose activation could occur through the plasminogen activation system.

### Galardin blocks EDP-stimulated B16F1 cell invasion by blocking MMPs activity

**Figure 3:**
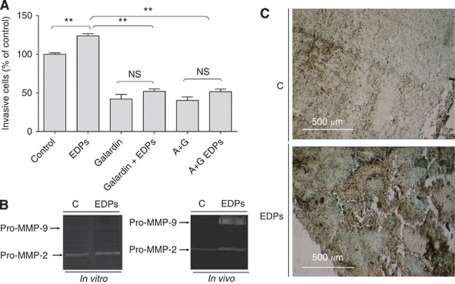
Elastin-derived peptide-stimulated B16F1 invasion involves the MMP system (**A**) without upregulating MMP-2 and MMP-9 expression and/or activation (**B**). EDPs increase *in vivo* infiltrating cells in B16F1 tumours (**C**). (**A**) Cellular invasive potential was assayed using Transwell coated with Matrigel (40 *μ*g per well). In total, 50 × 10^3^ cells in 100 *μ*l of RPMI 1640 with or without EDPs (50 *μ*g ml^–1^) were deposited in the upper compartment, in the presence or absence of galardin (10^−9^M, G), aprotinin (100 *μ*g ml^–1^, A) or both (A+G). The lower chamber contained 10% FBS and 2% of BSA. Incubation time was 40 h. Data are expressed as mean±s.e.m. values from three independent experiments, each performed in triplicate. ^**^Significantly different at *P*<0.01. (**B**) Gelatin zymography was performed as described in the Materials and Methods section. The gels presented are representative of several *in vitro* (*n*=3) and *in vivo* (*n*=5) experiments. (**C**) Infiltrating cells in B16F1 and EDP-treated B16F1 tumours were identified by immunohistochemistry. Tumour sections were stained with an anti-monocyte/macrophage antibody. Monocyte/macrophage appears as dark-brown staining. Tumour sections were counterstained with methylene green zinc chloride double salt. The photographs presented are representative of several (*n*=5) experiments.

The MMPs constitute the second major proteolytic system used by melanoma cells to invade ECM (Hofmann *et al*, 2000). The invasive properties of B16F1 and EDP-stimulated B16F1 cells were assayed *in vitro* in the presence of galardin (Agren *et al*, 2001). Galardin decreased invasion of B16F1 cells stimulated or not with EDPs (Figure 3A), suggesting that MMPs are required for basal and EDP-induced B16F1 invasion. Importantly, co-treatment of cells with galardin and aprotinin together (Figure 3A) gave the same results than galardin alone, indicating that MMPs activity is sufficient to mediate matrix degradation induced by EDPs. Owing to the important role of gelatinases A and B in the invasion process of melanoma (Hofmann *et al*, 2000; Seiki, 2002), we investigated their expression and their activation *in vitro* and *in vivo*. These analyses evidenced no variation in the pattern of expression and activation of these MMPs (Figure 3B), as well as for MT1-MMP (data not shown). Nevertheless, a high expression of pro-MMP9 was observed in EDP-treated tumours lysates.

### Increase in pro-MMP-9 expression in EDP-treated B16F1 tumours correlates with infiltration of monocytes/macrophages

The results described above let us to hypothesise that EDP-treated mice tumours contained more infiltrating monocytes/macrophages than controls. Indeed, it has been shown in tumours that MMP-9 is mostly expressed by these cells (Foda and Zucker, 2001). Therefore, we performed an immunolabelling of infiltrating monocytes/macrophages in tumour sections by using an anti-MoMa-2 antibody. Immunohistochemistry evidenced that infiltrating cells were highly present in EDP-treated tumours (Figure 3C).

### EDPs treatment increases Mcol-A secretion and activation through the plasminogen system

**Figure 4:**
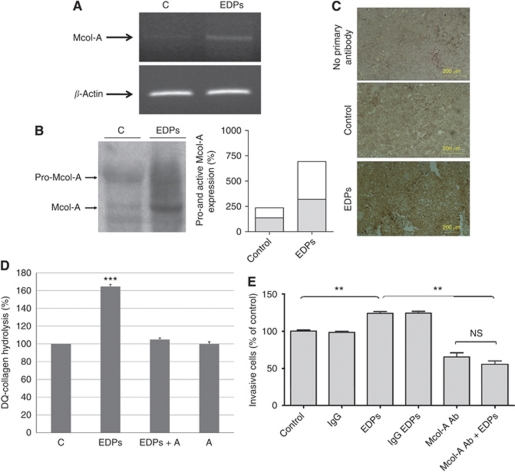
Elastin-derived peptides increase *in vitro* and *in vivo* Mcol-A expression and activation (**A**–**C**), leading to collagen degradation (**D**). Treatment of cells with anti-MMP-1-blocking antibody blocks EDP-stimulated B16F1 invasion (**E**). (**A**) Mcol-A expression was analysed by RT–PCR. B16F1 cells were treated with EDPs (50 *μ*g ml^–1^) for 48 h and RT–PCR was conducted as described in the Material and Methods section. The gel presented is representative of several (*n*=3) experiments. (**B**) Left panel: Mcol-A secretion and activation by B16F1 and EDP-treated B16F1 cells was analysed by western blotting using an anti-MMP-1 antibody as described in the Materials and Methods section. The gel presented is representative of several (*n*=3) experiments. Right panel: Densitometric analyses of western blots. White bars: active form; black bars: pro-form. (**C**) Mcol-A expression by tumours from B16F1- and EDP-treated B16F1 was analysed by immunohistochemistry. Tumour sections were stained with an anti-Mcol-A antibody. Mcol-A appears as dark-brown staining. Tumour sections were counterstained with hematoxylin. Scale bar=200 *μ*m. (**D**) B16F1-conditioned media (48 h) incubated or not with EDPs (50 *μ*g ml^–1^) and/or aprotinin (100 *μ*g ml^–1^) were incubated with DQ-collagen. The degradation of DQ-collagen was evaluated by fluorescence (*λ*_ex_=495 nm; *λ*_em_=515 nm). ^***^Significantly different at *P*<0.001. (**E**) Cellular invasive potential was assayed using Transwell coated with Matrigel (40 *μ*g per well). In total, 50 × 10^3^ cells were suspended in 100 *μ*l of RPMI 1640 with or without EDPs (50 *μ*g ml^–1^) in the upper compartment and in the presence or not of anti-MMP-1-blocking antibody (10 *μ*g ml^–1^). The lower chamber contained 10% FBS and 2% of BSA. Incubation was for 40 h. Data are expressed as mean±s.e.m. values from three independent experiments, each performed in triplicate. ^**^Significantly different at *P*<0.01. NS=nonsignificantly different.

Collagenase-1 (MMP-1) has been shown to be important in melanoma development (Walker and Woolley, 1999; Hofmann *et al*, 2005) and could be activated by the plasminogen system (Benbow *et al*, 1999). Additionally, it is known that EDPs strongly induce MMP-1 expression (Brassart *et al*, 2001). Reverse transcription PCR analysis (Figure 4A) demonstrated that EDPs significantly enhanced the expression of Mcol-A, the murine counterpart of MMP-1 (Balbin *et al*, 2001) comparatively to control cells. At the protein level, we found that EDPs increase the Mcol-A expression level but also its activation (Figure 4B). Moreover, data obtained from immunohistochemistry labelling confirmed these results by showing a strong increase of the expression of Mcol-A in EDP-treated mice tumours compared with control B16F1 tumours (Figure 4C).

In order to evaluate the consequence of this process on the degradation of the ECM and the role of the plasminogen system in Mcol-A activation, we measured the degradation of fuorigenic DQ-collagen by B16F1-conditioned media (Figure 4D). We show that EDPs drastically increased DQ-collagen degradation. The presence of aprotinin blocked this process. EDPs consequently trigger matrix degradation through collagen digestion and this process is dependent on Mcol-A activation by the plasminogen system.

### An anti-MMP-1 antibody blocks the effects of EDPs on melanoma invasion *in vitro*

In order to validate the involvement of Mcol-A in EDP-induced melanoma invasion, B16F1 cells treated or not with EDPs were preincubated with a specific blocking anti-MMP-1 antibody (Brennan *et al*, 2003) (10 *μ*g ml^–1^) and the invasiveness was assayed *in vitro.* As shown in Figure 4E, treatment of B16F1 and EDP-treated B16F1 with this antibody significantly decreased their invasive properties, reproducing the effect of galardin on B16F1 invasiveness after stimulation by EDPs. These results show that Mcol-A has a central role in elastin peptide-driven melanoma invasion.

## Discussion

The tumour microenvironment has an important role in cancer progression and *in vitro* assays have shown that matrix-derived peptides could be the important factors (Hornebeck *et al*, 2002). For instance, EDPs have been suggested to actively participate to melanoma growth and invasion. Nevertheless, no *in vivo* studies had been conducted to address this point.

We show for the first time, that EDPs are crucial modulators of melanoma growth *in vivo*. Moreover, we further suggest that Mcol-A could have a central role in EDP-enhanced tumour invasion.

Using a subcutaneous system of B16F1 implantation, we show that EDPs increase B16F1 tumour development 10 days post-challenge by three folds. We also demonstrated that EDPs induced *in vitro* proliferation of melanoma cells, demonstrating that they directly trigger this process *in vivo*. Such an effect has already been reported for several cell types (Duca *et al*, 2004). Our results comfort these data. Intense fragmentation of elastin has been observed at the invasive front of melanoma and *in vitro* studies suggested that EDPs could participate to such a process *in vivo* by inducing proteolytic cascades (Yusa *et al*, 1989; Timar *et al*, 1991; Svitkina and Parsons, 1993; Ntayi *et al*, 2004). However, no *in vivo* studies were previously carried out to confirm this hypothesis. We then analysed EDPs effect on melanoma migration and invasion using well-established *in vitro* migration and invasion systems. We have shown that EDPs increase the melanoma migration and also, by 36%, the B16F1 invasion. Altogether, these results demonstrate that EDPs enhanced melanoma growth and invasion both *in vitro* and *in vivo.*

Two main proteolytic cascades are involved in melanoma development, the plasminogen activation system and the MMPs family (Pasco *et al*, 2004a). We found that aprotinin blocked EDP-enhanced B16F1 invasion, suggesting a requirement of the plasminogen activation system. Surprisingly, we observed that this treatment did not induce neither tPA nor uPA expression *in vitro* nor *in vivo*. Moreover, we have shown that EDPs treatment does not modulate plasminogen activation (data not shown). This is in contrast with our results using aprotinin suggesting that this system is required in EDP-induced invasion. Therefore, even if tPA or uPA expressions are not induced, their basal activity level could be required to induce cell invasion by activating a novel pool of MMPs expressed following EDPs treatment. The analysis of the influence of galardin on EDPs-stimulated melanoma invasion demonstrates a blockade of the basal invasion of B16F1 cells as well as that induced by EDPs treatment. These results demonstrate a role of MMPs in the EDP-triggered invasion. Moreover, the fact that the use of aprotinin with galardin exhibited the same effects than galardin alone supports our previous hypothesis that MMPs constitute the central proteolytic system involved in EDP-enhanced melanoma invasion.

EDPs were shown to induce the expression of several MMPs in various tumour cell types (Duca *et al*, 2004). Among these proteases, MMP-2 has been largely involved in melanoma invasion (Ray and Stetler-Stevenson, 1995; Vaisanen *et al*, 1996) and a correlation between its expression and melanoma prognosis has been established (Vaisanen *et al*, 1998). Although conflicting data have been reported concerning the role of MMP-9 in melanoma invasion, several studies have shown that this MMP could be involved in the progression of this disease (Itoh *et al*, 1999; MacDougall *et al*, 1999). Although EDPs treatment had no effect on MMP-2 expression both *in vitro* and *in vivo*, a high increase in pro-MMP-9 expression *in vivo* was observed. However, as only the pro-form of this enzyme can be detected, its participation in matrix degradation could be excluded. MMP-9 can be produced by cancer or stromal cells but is mostly secreted by macrophages (Foda and Zucker, 2001). Moreover, previous *in vitro* studies showed that EDPs are chemotactic for inflammatory cells (Duca *et al*, 2004).

It was therefore possible that EDP-treated tumours contained more monocytes/macrophages than control tumours, which would explain the observed increase of pro-MMP-9 secretion. Immunohistochemistry experiments showed that EDP-treated tumours effectively exhibited a drastic increase in infiltrating monocytes/macrophages, confirming our hypothesis.

Many studies underline that MT1-MMP exhibits intrinsic matrix-degrading activities on various matrix proteins and promotes tumour invasion and melanoma growth (d’Ortho *et al*, 1997; Deryugina *et al*, 2002; Iida *et al*, 2004). We checked the influence of EDPs on MT1-MMP expression and no enhancement in MT1-MMP expression or activation, both *in vitro* and *in vivo*, was observed (data not shown).

Finally, MMP-1 was shown to be expressed during the invasion of malignant melanomas (Airola *et al*, 1999). In addition, high expression of MMP-1 was correlated with shorter disease-free survival in metastatic melanoma (Nikkola *et al*, 2002). Moreover, this enzyme can be activated by the plasminogen system (Benbow *et al*, 1999). EDPs treatment triggered a high increase of the murine counterpart of MMP-1, Mcol-A at the mRNA and at the protein level. Moreover, we showed that the plasminogen system is actually involved in the activation of Mcol-A, reinforcing our hypothesis. The use of DQ-collagen permitted us to demonstrate that this process triggers collagen degradation, explaining the influence of EDPs on B16F1 invasion. The increase in Mcol-A secretion was moreover demonstrated at the tissue level. Surprisingly, our immunolabelling also suggest the presence of Mcol-A in the nucleus. Such a colocalisation has also been reported for MIO-M1 cells, Tenon's fibroblasts and ARPE cells. Such localisation is thought to confer resistance to apoptosis and may explain the well-known association of this enzyme with tumour cell survival and spreading (Limb *et al*, 2005).

Our results strongly suggested that Mcol-A could be a key MMP by which EDPs exhibit their effects on melanoma invasion. To check this hypothesis, we used an anti-MMP-1 antibody directed against the active site of human MMP-1 and previously used in blocking experiments (Brennan *et al*, 2003). As the catalytic site of Mcol-A possesses 63% homology with human MMP-1 (Balbin *et al*, 2001), we assumed that this antibody could be used to block Mcol-A activity. As we hypothesised, the antibody inhibited EDPs effect on B16F1 cell invasion with the same efficiency than galardin, showing that Mcol-A is the key MMP involved in EDP-induced melanoma invasion.

It has already been suggested that MMP-1 could be important in melanoma development through its secretion by melanoma but also by stromal cells (Woolley and Grafton, 1980; Airola *et al*, 1999) and, recently, it was shown that shRNA-driven MMP-1 knockdown inhibited metastasis formation in a xenograft model (Blackburn *et al*, 2007). In our model, EDPs induced B16F1 invasion through Mcol-A expression, reinforcing the fact that MMP-1 is a crucial protease for melanoma invasion. However, its role in melanoma invasion is not as documented as that of the MT1-MMP/MMP-2 system. Importantly, despite our *in vitro* results, we could not affirm that Mcol-A production was limited to melanoma cells. Indeed, some infiltrating and/or stromal cells could produce this MMP and thereby participate to B16F1 invasion.

In conclusion, we propose that elastolysis could be a leading phenomenon in melanoma invasion *in vivo* because it promotes local collagenolysis. Previous *in vitro* studies suggesting that the stromal reaction induced by EDPs could promote collagen degradation through collagenase production (Brassart *et al*, 2001) supports this view. This could explain the histopathological data underlining a strong correlation between elastin fragmentation and melanoma aggressiveness (Breslow, 1970; Feinmesser *et al*, 2002). Moreover, it was proposed that EDPs could act as factors promoting the transition between melanoma radial growth phase to vertical growth phase in which melanoma cells infiltrate and invade the dermis. Interestingly, this transition is associated with a proteolytic switch in which MMP-1 expression is increased (Hornebeck *et al*, 2005; Antonicelli *et al*, 2007). Our study therefore reinforced this hypothesis.

It was suggested that partial preservation of elastic fibres in the tumour depth was a relatively good predictive factor, whereas complete absence of elastin was an adverse one (Feinmesser *et al*, 2002). However, this study was based on histological analyses and no *in vivo* studies were carried out to confirm this hypothesis. Consequently, owing to the lack of *in vivo* demonstration of the active role of EDPs in melanoma growth, this point was underconsidered. Our results demonstrating that EDPs drastically enhanced melanoma growth and invasion reinforced this assumption. Consequently, we confirm and demonstrate that elastin degradation could be an important predictive factor of melanoma evolution.
